# A real-world study comparing perioperative chemotherapy and EGFR-tyrosine kinase inhibitors for treatment of resected stage III *EGFR*-mutant adenocarcinoma

**DOI:** 10.1186/s12885-023-11342-y

**Published:** 2023-09-11

**Authors:** Chieh-Lung Chen, Sing-Ting Wang, Wei-Chih Liao, Chia-Hung Chen, Chih-Yen Tu, Te-Chun Hsia, Wen-Chien Cheng, Hung-Jen Chen

**Affiliations:** 1 Division of Pulmonary and Critical Care, Department of Internal Medicine, China Medical University Hospital, No. 2, Yude Road, North District, Taichung City, 404327 Taiwan; 2 Division of Hematology and Oncology, Department of Internal Medicine, China Medical University Hospital, Taichung, 404327 Taiwan; 3 School of Medicine, College of Medicine, China Medical University, Taichung, 404333 Taiwan; 4 Department of Life Science, National Chung Hsing University, Taichung, 40227 Taiwan; 5 Ph.D. Program in Translational Medicine, National Chung Hsing University, Taichung, 40227 Taiwan; 6 Rong Hsing Research Center for Translational Medicine, National Chung Hsing University, Taichung, 40227 Taiwan

**Keywords:** Adenocarcinoma, Chemotherapy, Epidermal growth factor receptor (EGFR), Stage III, Surgery, Tyrosine kinase inhibitor (TKI)

## Abstract

**Background:**

The patient population with stage III non-small-cell lung cancer (NSCLC) is heterogeneous, with varying staging characteristics and diverse treatment options. Despite the potential practice-changing implications of randomized controlled trials evaluating the efficacy of perioperative epidermal growth factor receptor-tyrosine kinase inhibitors (EGFR-TKIs), concerns have been raised due to conflicting overall survival (OS) results. Few real-world studies have examined the survival outcomes of patients with resected *EGFR*-mutant stage III adenocarcinoma receiving perioperative chemotherapy and EGFR–TKIs.

**Methods:**

In this retrospective observational study, we enrolled patients with resected stage III adenocarcinoma with *EGFR* mutations between January 2011 and December 2021. Patients were classified into two groups: perioperative chemotherapy and perioperative EGFR–TKIs. Outcomes and prognostic factors were analyzed using Cox proportional hazards regression analysis.

**Results:**

Eighty-four patients were enrolled in the analysis. Perioperative EGFR-TKIs led to longer progression-free survival (PFS) than chemotherapy (38.6 versus 14.2 months; p = 0.019). However, only pathological risk factors predicted poor PFS in multivariate analysis. Patients receiving perioperative chemotherapy had longer OS than those receiving EGFR-TKIs (111.3 versus 50.2 months; p = 0.052). Multivariate analysis identified perioperative treatment with EGFR-TKIs as an independent predictor of poor OS (HR: 3.76; 95% CI: 1.22–11.54).

**Conclusion:**

Our study demonstrates that chemotherapy should be considered in the perioperative setting for high-risk patients, when taking pathological risk factors into consideration, and that optimized sequencing of EGFR–TKIs might be the most critical determinant of OS.

## Background

The patient population with stage III non-small-cell lung cancer (NSCLC) is heterogeneous, with varying staging characteristics and diverse treatment options, including surgery, systemic therapy, and concurrent systemic and radiation therapy. Although surgery offers the best chances of long-term survival to patients with primary NSCLC [1], only 30% of stage III NSCLC tumors are resectable [2]. Neoadjuvant therapy is a widely accepted approach for treating patients with stage III lung cancer, as it effectively downstages NSCLC and increases the probability of successful curative surgery. Studies have shown that neoadjuvant chemotherapy can offer an overall survival (OS) advantage of up to 4% compared to surgery alone [3]. Additionally, cisplatin-based adjuvant chemotherapy is considered the standard of care for resected stage III NSCLC due to its proven OS benefit [4]. A meta-analysis indirectly compared the effects of adjuvant and neoadjuvant chemotherapy on survival and found them to be similar [5].

Adenocarcinoma accounts for approximately 50–60% of all stage III NSCLC in Asian populations [6, 7]. In these populations, the epidermal growth factor receptor (*EGFR*) mutation has been identified in 50–60% of patients [6]. The *EGFR* mutation is associated with a higher risk of metastatic recurrence in locally advanced stage III adenocarcinoma [8]. Accumulating data show the efficacy of adjuvant EGFR–tyrosine kinase inhibitors (EGFR–TKIs) for treating patients with resected *EGFR*-mutant NSCLC [9–11].

The ADJUVANT study demonstrated that adjuvant gefitinib resulted in a significantly longer disease-free survival (DFS) than cisplatin plus vinorelbine in patients with completely resected stage II–IIIA *EGFR*-mutant NSCLC [10]. However, this DFS advantage did not translate to a significant difference in OS [12]. A meta-analysis concluded that adjuvant EGFR–TKI therapy for resected *EGFR*-mutant NSCLC significantly improves DFS but not OS [13]. In the ADAURA study, patients with completely resected stage IB–IIIA *EGFR*-mutant NSCLC receiving the adjuvant osimertinib showed significantly longer DFS than those receiving a placebo, and the hazard ratio (HR)(0.12; 95% CI: 0.07–0.2) of patients with stage IIIA disease remained significantly lower [11]. Despite these promising results, the ADAURA trial was not designed to compare the efficacy of adjuvant chemotherapy and adjuvant EGFR-TKIs. Evidence supporting the feasibility of neoadjuvant EGFR-TKIs in the perioperative setting has also been provided by the EMERGING-CTONG1103 and NeoADAURA trials [14, 15].

Based on these studies, perioperative (neoadjuvant and/or adjuvant) chemotherapy and EGFR-TKIs provided a survival benefit in early-stage NSCLC. Despite many studies evaluating the role of perioperative EGFR–TKIs, the patient pool with stages IB–IIIA represents a very wide variety of cancers with inconstant prognosis [16], which may limit the application of these results to resected stage III *EGFR*-mutant adenocarcinoma. Currently, there are few real-world studies examining the survival outcomes of patients receiving perioperative chemotherapy and EGFR–TKIs for resected *EGFR*-mutant stage III adenocarcinoma [17]. The prognostic effect of pathological factors has never been emphasized in previous randomized controlled trials (RCTs). This retrospective study aimed to compare the treatment outcomes, with a focus on OS, between patients with resected *EGFR*-mutant stage III adenocarcinoma who received either perioperative chemotherapy or EGFR-TKIs.

## Materials and methods

### Study design and patients

This retrospective study investigated patients with resected stage III (according to American Joint Committee on Cancer, 8th edition) [18] *EGFR*–mutant adenocarcinoma at a tertiary referral center in Taiwan between January 2011 and December 2021. The study adhered to the Declaration of Helsinki and followed the STROBE guidelines for reporting observational studies. The Institutional Review Board of China Medical University Hospital (IRB number: CMUH110-REC1-244) waived the need for informed consent from study subjects due to the retrospective design.

The study collected and recorded data on the baseline characteristics of each patient, which included sex, age, smoking status, Eastern Cooperative Oncology Group Performance Status (ECOG-PS), tumor-node-metastasis (TNM) stage, *EGFR* mutation subtype, perioperative antineoplastic therapy, and subsequent antineoplastic therapy after disease progression.

### Treatment exposure

As routine clinical practice, the treatment for each patient was discussed by the multidisciplinary team. Patients who received surgery and either neoadjuvant or adjuvant antineoplastic therapy were included in our study. The surgical procedures were decided by individual surgeons according to the size and location of the tumors. Based on the perioperative treatment regimen, the patients were classified into two groups: the perioperative chemotherapy group and the perioperative EGFR–TKI group. During the study period, the use of neoadjuvant EGFR-TKIs was an off-label treatment, and patients were given the choice to receive the treatment after a thorough explanation from their physician. Decisions about whether patients would receive radiotherapy were made by the physician.

### Pathological examination

Tissue slides stained with the hematoxylin–eosin stain, immunohistochemistry stain, or elastic stain were reviewed by experienced pathologists. The presence of tumor cells in the lymphatic or vascular lumen, the space around nerves, or the visceral pleura was defined as lymphovascular invasion [19], perineural invasion [20, 21], or visceral pleural invasion [22], respectively. Margin involvement was defined as microscopic residual disease at the resection margin. The extension of malignant cells through the nodal capsule was considered an extranodal extension. The above-mentioned characteristics from the pathological examination were defined as “pathological risk factors.”

### Clinical assessments and efficacy evaluations

At baseline, patients underwent imaging studies including chest computed tomography (CT), brain magnetic resonance imaging, and positron emission tomography to determine the stage of the disease and evaluate any metastasis. In selected patients, endobronchial-ultrasound transbronchial needle aspiration (EBUS-TBNA) was performed for N staging based on the multidisciplinary team’s suggestion.

All patients received chest CT evaluations every 12 weeks to evaluate tumor response after initiation of antineoplastic therapy. Other images were obtained when suspicious new symptoms developed. Progression-free survival (PFS) was the time elapsed between the date of initiation of stage III NSCLC treatment and radiological progression (according to the Response Evaluation Criteria in Solid Tumors v1.1), clinical progression, or death. OS was the time elapsed between the date of diagnosis and death. In cases where disease progression or death were not recorded, patients were censored either at the end of the observation period, which was 30 June 2022, or at the time of their last available medical record entry.

### Statistical analyses

Statistical analyses were conducted with MedCalc for Windows version 18.10 (MedCalc Software, Ostend, Belgium). For normally distributed variables, the mean ± standard deviation was used, while the median and interquartile range were used for non-normally distributed variables. *t*-tests were used to analyze continuous data with normal distributions, while categorical variables were presented as percentages and numbers, and analyzed with either the chi-square or Fisher’s exact test. The Kaplan-Meier method was used to evaluate PFS and OS, while Cox proportional hazards regression analysis was used to analyze prognostic factors. The HR of disease progression and mortality was calculated using univariate analysis, and the multivariate regression model included significant variables from univariate analysis and clinically important variables to adjust potential confounders. The strength of the association was presented as the HR and its 95% confidence interval (CI). A *p*-value of < 0.05 was considered statistically significant.

## Results

**Fig. 1:**
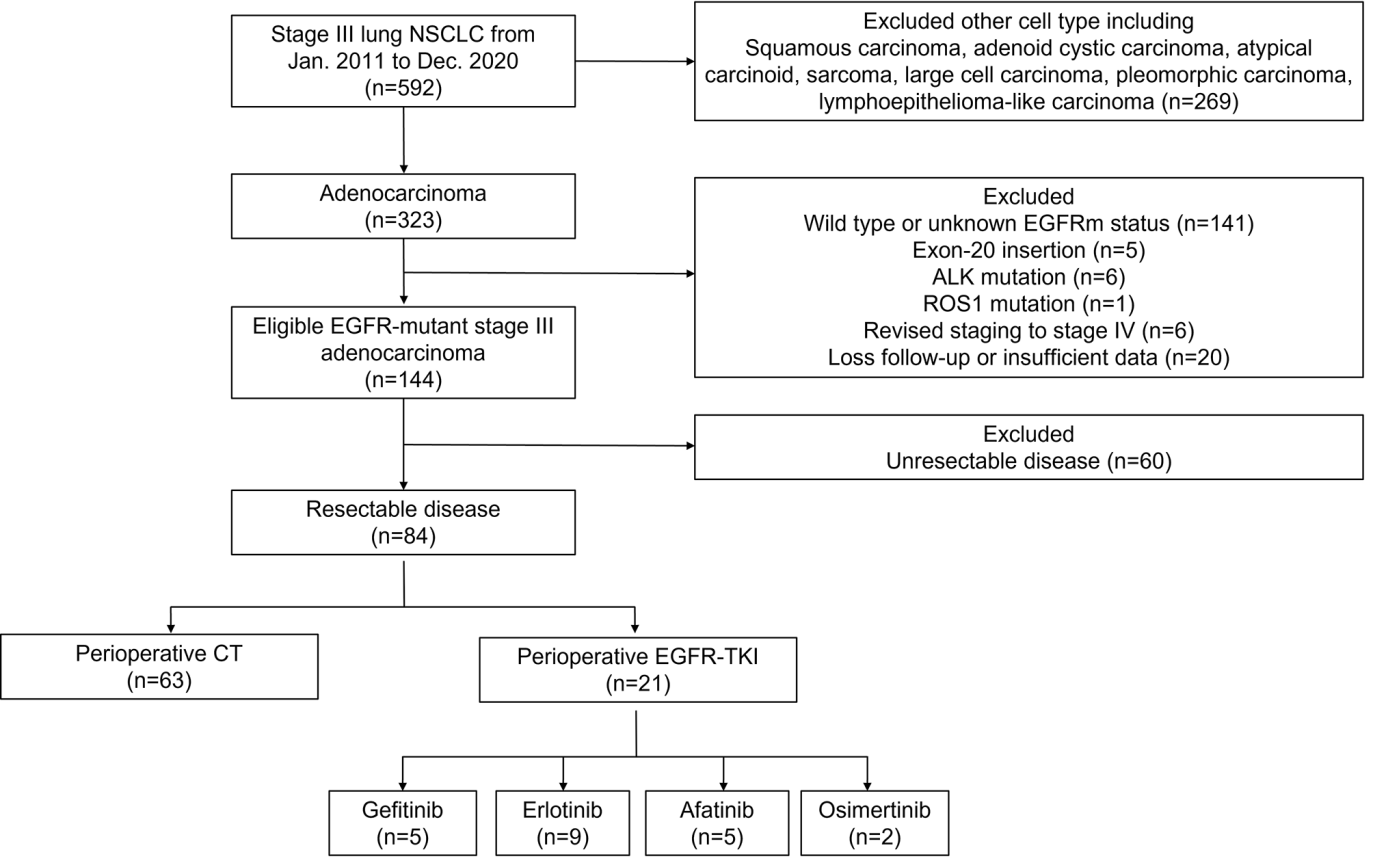
Flowchart showing patient selection. ALK: anaplastic lymphoma kinase; CT: chemotherapy; EGFR, epidermal growth factor receptor; NSCLC, non-small-cell lung cancer; TKI, tyrosine kinase inhibitor

Eighty-four patients with resected stage III *EGFR*-mutant adenocarcinoma were enrolled in this study. Sixty-three patients received perioperative chemotherapy and 21 received perioperative EGFR–TKIs (Fig. 1). Of the patients receiving perioperative EGFR–TKIs, nine were treated with erlotinib, five with gefitinib, five with afatinib, and two with osimertinib.

Patients receiving perioperative EGFR–TKIs were older than those receiving chemotherapy (61.9% of patients in the EGFR–TKI group were ≥ 65 years old versus 30.2% in the chemotherapy group, *p* = 0.009). No significant differences in gender, ECOG–PS, smoking status, and the *EGFR* mutation were observed between groups (Table 1). More patients in the EGFR–TKI group received neoadjuvant treatment (38.1% versus 9.5%, *p* = 0.002).

**Table 1:** Clinical characteristics of the patients with resected *EGFR*–mutant stage III adenocarcinoma in this study

		All (N = 84)	Perioperative chemotherapy (N = 63)	Perioperative EGFR–TKI (N = 21)	*p*-value
Age ≥ 65 years		32 (38.1%)	19 (30.2%)	13 (61.9%)	0.009
Male		27 (32.1%)	20 (31.7%)	7 (33.3%)	0.893
Smoking		21 (25%)	17 (27%)	4 (19%)	0.467
ECOG-PS					0.250
	0–1	83 (98.8%)	63 (100%)	20 (95.2%)	
	≥ 2	1 (1.2%)	0	1 (4.8%)	
EGFR mutation					0.933
	Del 19	37 (44%)	27 (42.9%)	10 (47.6%)	
	L858R	42 (50%)	31 (49.2%)	11 (52.4%)	
	Uncommon mutations^a^	5 (6%)	5 (7.9%)	0	
Perioperative treatment					0.002
Neoadjuvant^b^		14 (16.7%)	6 (9.5%)	8 (38.1%)	
Adjuvant		70 (83.3%)	57 (90.5%)	13 (61.9%)	
Pathology results					
	Lymphovascular or perineural invasion	60 (71.4%)	50 (79.4%)	10 (47.6%)	0.005
	Pleural invasion	47 (56%)	40 (63.5%)	7 (33.3%)	0.016
	Margin involvement	5 (6%)	3 (4.8%)	2 (9.5%)	0.595
	Extranodal extension	26 (31%)	22 (34.9%)	4 (19%)	0.275
Lymph node status					0.100
	N0–1	14 (16.7%)	10 (15.9%)	4 (19%)	
	N2	69 (82.1%)	53 (84.1%)	17 (81%)	
EGFR–TKI					
	Perioperative		0	21 (100%)	
	Subsequent		52 (82.5%)	7 (33.3%)	

ECOG-PS, Eastern Cooperative Oncology Group Performance Status; EGFR, epidermal growth factor receptor; OP: operation; TKI, tyrosine kinase inhibitor.

a: Exon-20 insertion was excluded. Uncommon EGFR mutations were detected in 2 (G719X), 2 (L861Q), and 1 (G796V) patient. b: Among the 14 patients who underwent neoadjuvant treatment, 10 were observed postoperatively because of clinical downstaging. Out of these 10 patients, 5 were from the chemotherapy group and 5 were from the EGFR-TKI group.

**Fig. 2:**
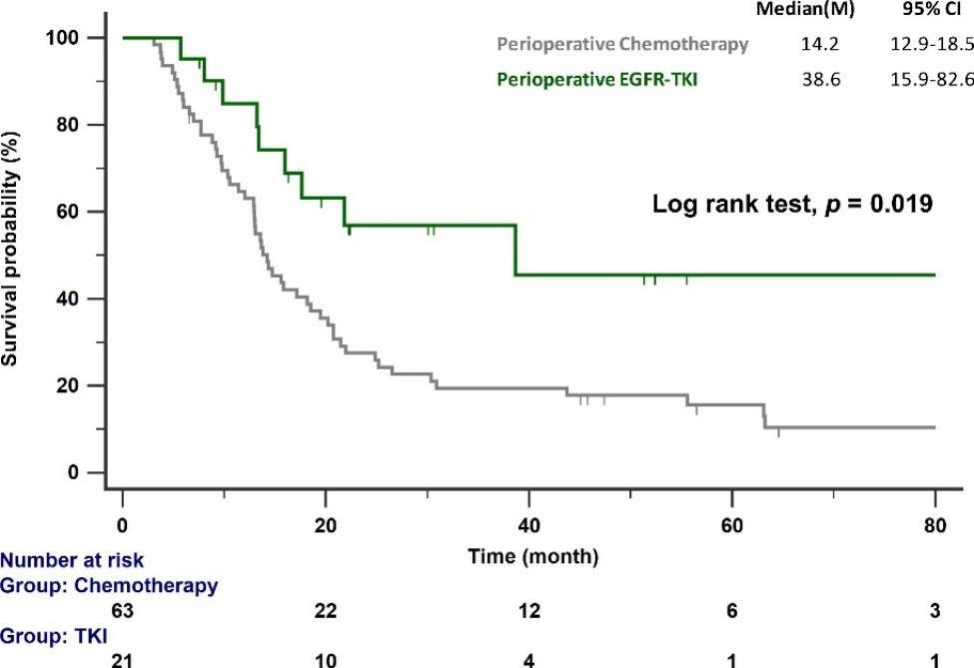
PFS of patients with *EGFR*-mutant NSCLC treated with perioperative chemotherapy and EGFR–TKIs. EGFR, epidermal growth factor receptor; PFS, progression-free survival; TKI, tyrosine kinase inhibitor

After a median follow-up of 53.2 months (range 45.5–61.0 months), 85.7% of patients in the perioperative chemotherapy group and 47.6% in the perioperative EGFR–TKI group experienced disease progression. The median PFS of patients receiving perioperative EGFR–TKIs was significantly longer than that of patients receiving perioperative chemotherapy (38.6 versus 14.2 months; *p* = 0.019; Fig. 2).

Multivariate Cox proportional hazards regression analysis was used to identify prognostic factors of poor PFS. The difference in HR between perioperative EGFR–TKIs and perioperative chemotherapy was not statistically significant. The presence of pathological risk factors was an independent prognosticator of poor PFS (HR: 2.36; 95% CI: 1.14–4.91) (Table 2).

**Table 2:** Cox proportional hazards regression analysis of the PFS of patients with resected *EGFR*–mutant stage III adenocarcinoma

	Univariate model			Multivariate model		
	HR	95% CI	*p*-value	HR	95% CI	*p*-value
Age ≥ 65 years	0.65	0.38–1.10	0.106	0.74	0.43–1.28	0.280
Male	1.29	0.76–2.19	0.356			
Smoking	1.50	0.87–2.59	0.150	1.73	0.97–3.08	0.064
L858R versus Del 19 mutation	0.97	0.58–1.60	0.897			
T4	0.88	0.45–1.72	0.700			
N2	1.13	0.57–2.24	0.733			
Perioperative EGFR–TKI versus chemotherapy	0.46	0.23–0.90	0.023	0.71	0.34–1.48	0.362
Pathological risk factors^a^	2.56	1.30–5.06	0.007	2.36	1.14–4.91	0.021

CI: Confidence interval; EGFR, epidermal growth factor receptor; HR: hazard ratio; PFS, progression-free survival; TKI, tyrosine kinase inhibitor

a: The presence of any of the following: lymphovascular invasion, perineural invasion, pleural invasion, and margin involvement

**Fig. 3:**
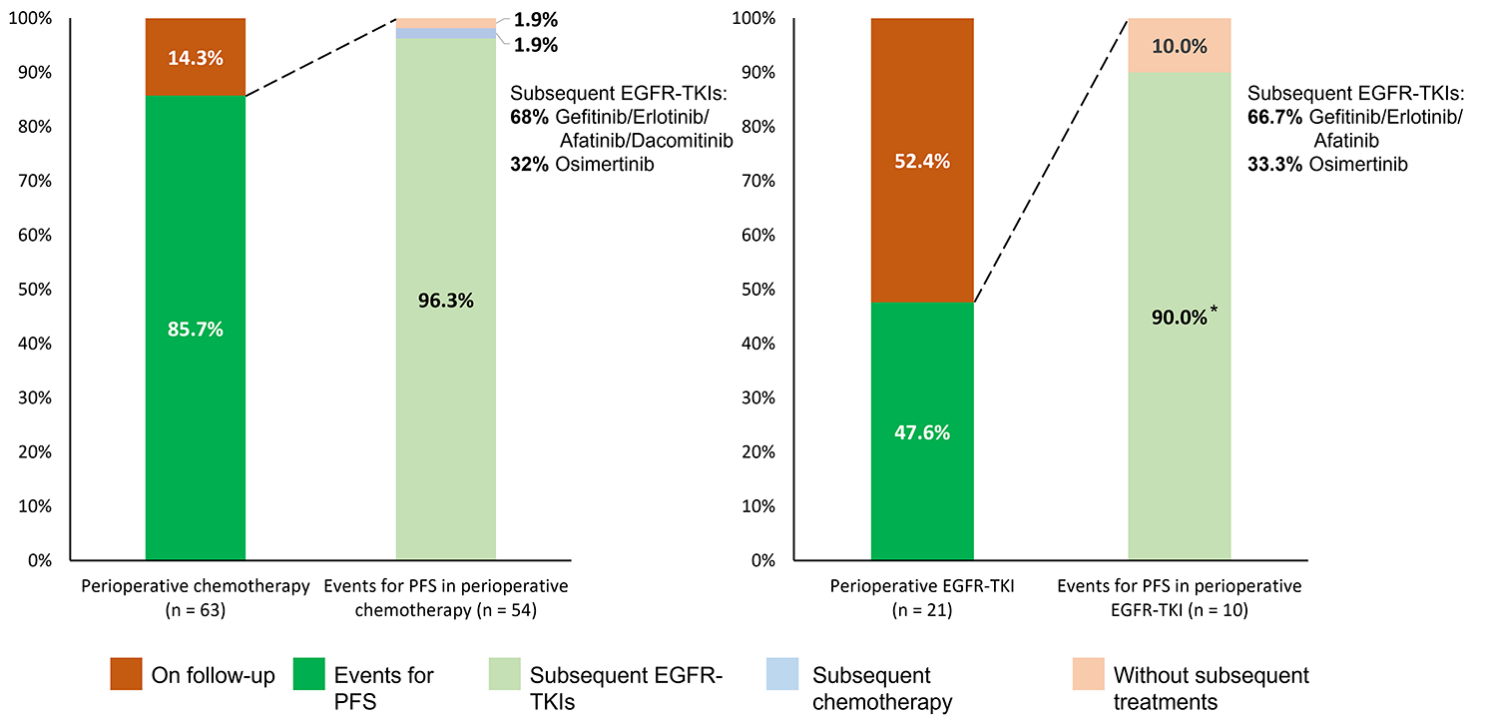
Subsequent treatment regimens of patients treated with perioperative chemotherapy or EGFR–TKIs. EGFR, epidermal growth factor receptor; PFS, progression-free survival; TKI, tyrosine kinase inhibitor. *Nine of the ten patients with disease progression in the perioperative EGFR-TKI group received EGFR-TKI treatment, while only four received chemotherapy

In the case of patients experiencing disease progression, 96.3% (52/54) in the perioperative chemotherapy group and 90% (9/10) in the perioperative EGFR–TKI group received subsequent EGFR–TKI treatment (Fig. 3). Of these patients, 32% (16/50) in the chemotherapy group and 33.3% (3/9) in the EGFR–TKI group received osimertinib as the later-line treatment. Of the 10 patients with disease progression in the perioperative EGFR–TKI group, only four received chemotherapy as their subsequent treatment (Fig. 3).

**Fig. 4:**
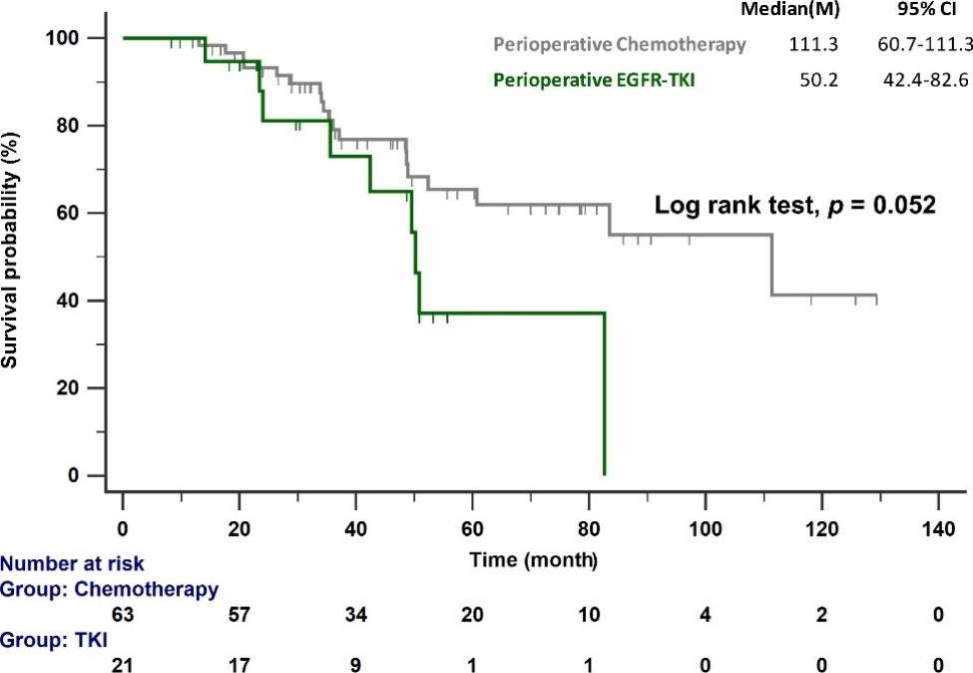
OS of patients with *EGFR*-mutant NSCLC treated with perioperative chemotherapy and *EGFR*–TKI. *EGFR*, epidermal growth factor receptor; OS, overall survival; TKI, tyrosine kinase inhibitor

Twenty-eight (33.3%) deaths were recorded; 30.2% (19/63) of patients in the chemotherapy group and 42.9% (9/21) in the EGFR–TKI group died. The median OS of patients receiving perioperative chemotherapy was longer than that of patients receiving perioperative EGFR–TKIs (111.3 versus 50.2 months; *p* = 0.052; Fig. 4). Cox proportional hazards regression analysis to identify prognostic factors of poor OS revealed that treatment with perioperative EGFR–TKIs (HR: 3.76; 95% CI: 1.22–11.54) was an independent prognosticator of poor OS (Table 3). Although not statistically significant, a trend toward poor OS was observed in patients with a history of smoking (HR: 2.53; 95% CI: 1.00–6.48, *p* = 0.051).

**Table 3:** Cox proportional hazards regression analysis of the OS of patients with resected *EGFR*–mutant stage III adenocarcinoma

	Univariate model			Multivariate model		
	HR	95% CI	*p*-value	HR	95% CI	*p*-value
Age ≥ 65 years	1.80	0.84–3.87	0.131	1.47	0.61–3.53	0.394
Male	1.29	0.56–2.96	0.550			
Smoking	1.90	0.83–4.37	0.131	2.54	1.00–6.48	0.051
L858R versus Del 19 mutation	0.98	0.46–2.08	0.948			
T4	1.21	0.46–3.20	0.697	0.63	0.14–2.84	0.551
N2	1.17	0.44–3.12	0.751	0.95	0.24–3.71	0.936
Perioperative EGFR–TKI versus chemotherapy	2.20	0.97–4.98	0.058	3.76	1.22–11.54	0.021
Osimertinib	0.59	0.25–1.40	0.228			
Pathological risk factors^a^	1.20	0.45–3.18	0.711	2.36	0.81–6.86	0.114

CI: Confidence interval; EGFR, epidermal growth factor receptor; HR: hazard ratio; OS, overall survival; TKI, tyrosine kinase inhibitor.

a: The presence of any of the following: lymphovascular invasion, perineural invasion, pleural invasion, and margin involvement.

## Discussion

The heterogeneity of stage III NSCLC presents greater clinical complexity than patients enrolled in clinical trials. Neoadjuvant and/or adjuvant (perioperative) therapy is a frequently used treatment modality in clinical practice. Our study is the first real-world investigation to compare the impact of perioperative chemotherapy and EGFR-TKIs on the OS of patients with *EGFR*-mutant stage III NSCLC after incorporating pathological factors.

In this study, the median PFS of the perioperative chemotherapy group was 14.2 months and that of the perioperative EGFR–TKI group was 38.6 months. The PFS benefit of EGFR–TKIs in this patient population was similar to that of patients with advanced EGFR-mutant NSCLC [23–25]. However, the HR between perioperative EGFR–TKIs and chemotherapy was not statistically significant in the multivariate analysis. We found that the presence of pathological risk factors was an independent prognosticator of poor PFS (Table 2). The predictive effect of lymphovascular invasion on local–regional failure [19] and distant recurrence [26, 27] has been demonstrated before. Visceral pleural invasion [19, 22] and perineural invasion [20, 21] are also considered prognostic factors of poor PFS. Our results suggest that pathological features are critical and recognizing patients with higher risk of recurrence might facilitate the selection of adjuvant systemic treatments.

Despite the lower PFS associated with perioperative chemotherapy, OS was better due to this treatment. While more than 90% of patients in the chemotherapy group received EGFR–TKIs after disease progression, only 44.4% of patients in the EGFR–TKI group received chemotherapy as their subsequent treatment. This low crossover rate may be a factor contributing to the poorer OS seen in the EGFR group. Fewer patients in the EGFR–TKI group experienced disease progression (47.6% versus 85.7% in the chemotherapy group), but 90% (9/10) of them died. Although patients receiving perioperative EGFR–TKIs were older, the prognostic effect of age was adjusted in the multivariate analysis. The pathological examination showed that the EGFR–TKI group had fewer pathological risk factors than the chemotherapy group. However, adjuvant chemotherapy overcame the disadvantage to PFS and the poor pathological risk factors and contributed to better OS.

Accumulating evidence indicates the potential efficacy of neoadjuvant EGFR-TKIs in patients with resectable NSCLC, which has led to the design of RCTs, notably the phase II EMERGING-CTONG1103 and phase III NeoADAURA trials. Although the NeoADAURA trial was designed to evaluate the efficacy of neoadjuvant osimertinib with or without chemotherapy, adjuvant systemic treatment with either osimertinib or chemotherapy was allowed based on investigator choice for optimal care. In the EMERGING-CTONG 1103 trial, patients were divided into neoadjuvant/adjuvant erlotinib and chemotherapy groups. Both studies did not clearly distinguish between neoadjuvant and adjuvant treatments, leading to a more generalized “perioperative” treatment proposition [15, 28] as in the current study. The analysis of OS in the EMERGING-CTONG 1103 trial showed that, although there was a survival benefit in PFS, it did not translate into a difference in OS [28]. Moreover, this study was limited by the fact that only 69.7% of patients in the chemotherapy group received subsequent EGFR-TKI treatment after disease progression, which may confound the OS result.

Among previous RCTs evaluating the efficacy of adjuvant EGFR–TKIs, the EVAN phase II trial was the first to show a significantly higher OS benefit from erlotinib than from chemotherapy (vinorelbine plus cisplatin) in patients with resected stage IIIA *EGFR*-mutant NSCLC [29]. However, the sample size was relatively small (n = 51 in each group) and only 37.3% of the patients in the chemotherapy group received EGFR–TKIs after disease progression [30]. In our study, more than 90% of the patients in the perioperative chemotherapy group with disease progression received EGFR–TKIs. The negative OS outcomes reported in both the CTONG1104 and IMPACT trials have raised concerns about the potential for adjuvant targeted therapy to only delay disease recurrence rather than providing a cure [31, 32]. In the ADAURA trial, adjuvant osimertinib provided a significant OS benefit among patients with completely resected *EGFR*-mutant NSCLC [33]. However, only 43% of patients in the control arm received osimertinib as subsequent treatment after disease progression [33], which may bias the OS result. Based on the available evidence and the results of the current study, chemotherapy remains an essential component in perioperative settings for resected *EGFR*-mutant NSCLC patients [34].

Patients with a history of smoking tended to have worse OS outcomes in our study (HR 2.54; 95% CI 1.00–6.48; *p* = 0.051). For advanced NSCLC patients with *EGFR* mutations, smoking was associated with shorter PFS during EGFR–TKI treatment [35] and reduced OS [36, 37]. Smokers’ tumors are hypothesized to have a higher burden of alternative driver oncogene mutations and the likelihood of an escape mechanism [38]. In the subgroup analysis of the ADJUVANT study, the DFS benefit of gefitinib was non-significant for smokers (HR 0.56; 95% CI: 0.27–1.19). We regard smoking as a risk factor of reduced response to EGFR–TKIs; therefore, standard chemotherapy should be considered in the adjuvant setting for this patient group.

The NEJ009 study reported that patients with *EGFR*-mutant NSCLC could gain an OS benefit from combined treatment with chemotherapy and EGFR–TKIs compared to EGFR–TKIs alone [39]. In the ADAURA trial, subgroup analysis stratifying the benefit of adjuvant chemotherapy showed that the two-year DFS of patients who received adjuvant chemotherapy was better than that of the patients who did not receive this treatment (HR of 0.16 vs. 0.23) [40]. The present study also showed that perioperative chemotherapy with sequential EGFR–TKIs resulted in better OS. Subsequent therapy after disease progression also plays an important role in contributing OS.

Our study has several limitations. First, it was a retrospective, single-institution study, and the number of patients in our cohort was small. As a result, we were unable to divide patients who received perioperative systemic treatment into neoadjuvant or adjuvant treatment separately. However, currently available evidence suggests no difference in the survival of neoadjuvant or adjuvant chemotherapy [5]. Second, different types of EGFR-TKIs were analyzed together, and the effectiveness of a specific drug could not be evaluated. Third, there were imbalanced baseline characteristics between the two groups, and neoadjuvant treatment inevitably affected the pathology findings. However, due to the limited number of patients, we were unable to perform propensity score matching. To account for the influence of age, pathologic risk factors, T and N staging, and smoking history on OS, we utilized multivariate Cox proportional hazards regression analysis. Fourth, due to the retrospective nature of this study, the entry time varied across the patient population. Furthermore, only 9 cases with OS events were observed in the EGFR-TKI group, potentially leading to insufficient observation time for other patients and unavoidable bias. Fifth, excluding inoperable patients with neoadjuvant treatment may introduce selection bias. PFS was defined as the time from the initiation of antineoplastic treatment until disease progression in this study. For those undergoing neoadjuvant treatment followed by surgery, the combined treatment and surgical duration in PFS could lead to longer observed PFS times and potential immortal time bias. However, the patient’s neoadjuvant EGFR-TKI within three months before surgery ensures the immortal time bias is not significant, similar to neoadjuvant chemotherapy. Sixth, the result from this study should be interpreted cautiously in patients with uncommon mutations. All 5 patients with uncommon mutations received perioperative chemotherapy in our study. This bias was explained by the less active treatment effect of EGFR-TKIs in treating uncommon mutations, as compared with common mutations such as the del 19 mutation [41]. Finally, the percentage of patients using osimertinib after disease progression was relatively small (32% in the chemotherapy group and 33.3% in the EGFR–TKI group).

## Conclusion

Our study demonstrates that standard chemotherapy still should be considered in the perioperative setting for high-risk patients, when taking pathological risk factors into consideration, and that optimized sequencing of EGFR–TKIs might be the most critical determinant of OS in patients with stage III *EGFR*-mutant NSCLC.

## Data Availability

The datasets used and analyzed during the present study are available from the corresponding author on reasonable request.

## And acronyms

CI: Confidence interval
DFS: Disease free survival
EGFR: Epidermal growth factor receptor
EGFR-TKI: Epidermal growth factor receptor tyrosine kinase inhibitor
HR: Hazard ratio
NSCLC: Non-small cell lung cancer
OS: Overall survival
PFS: Progression-free survival
TKI: Tyrosine kinase inhibitor
